# A Diverse Family of Host-Defense Peptides (Piscidins) Exhibit Specialized Anti-Bacterial and Anti-Protozoal Activities in Fishes

**DOI:** 10.1371/journal.pone.0159423

**Published:** 2016-08-23

**Authors:** Scott A. Salger, Katherine R. Cassady, Benjamin J. Reading, Edward J. Noga

**Affiliations:** 1 Department of Applied Ecology, North Carolina State University, Raleigh, North Carolina, United States of America; 2 Department of Clinical Sciences, North Carolina State University, College of Veterinary Medicine, Raleigh, North Carolina, United States of America; Oklahoma State University, UNITED STATES

## Abstract

Conventional antibiotics and other chemical-based drugs are currently one of the most common methods used to control disease-related mortality in animal agriculture. Use of the innate immune system to decrease disease related mortalities is a novel alternative to conventional drugs. One component of the innate immune system is the host-defense peptides, also known as antimicrobial peptides. Host-defense peptides are typically small, amphipathic, α-helical peptides with a broad-spectrum of action against viral, bacterial, fungal, and/or protozoal pathogens. Piscidins are host-defense peptides first discovered in the hybrid striped bass (white bass, *Morone chrysops*, x striped bass, *M*. *saxatilis*). In this paper we identify four new piscidin isoforms in the hybrid striped bass and describe their tissue distributions. We also determine the progenitor species of origin of each piscidin (orthology) and propose a revised nomenclature for this newly described piscidin family based on a three class system. The Class I piscidins (22 amino acids in length; striped bass and white bass piscidin 1 and piscidin 3) show broad-spectrum activity against bacteria and ciliated protozoans, while the Class III piscidins (55 amino acids in length; striped bass and white bass piscidin 6 and striped bass piscidin 7) primarily show anti-protozoal activity. The Class II piscidins (44–46 amino acids in length; striped bass and white bass piscidin 4 and white bass piscidin 5) have a level of activity against bacteria and protozoans intermediate to Classes I and III. Knowledge of piscidin function and activity may help in the future development of disease-resistant lines of striped bass and white bass that could be used to produce superior hybrids for aquaculture.

## Introduction

Infectious diseases are a serious impediment to the success of any agricultural endeavor, including aquaculture, and can lead to significant economic losses [1–3]. It is estimated that disease-related mortality can approach 10% even in developed countries with good management practices for animal agriculture [4–5]. A primary means of controlling pathogens in animal agriculture includes the administration of drugs, principally antibiotics and other chemicals, which require a protracted and costly approval process. There are also public concerns about their use in food production and potential effects on the human population and the environment. These concerns include chemical residues left in the animal carcass that may be passed onto the consumer and the development of antibiotic-resistant microorganisms. Because of this, prophylactic vaccinations against important fish pathogens, as opposed to reactive drug treatments, are playing an increasingly important role in aquaculture [4,6–7]. Public safety concerns also have led to a shift from reactive drug use to other proactive strategies, such as promoting disease resistance through selective breeding. In order to carry out this proactive shift, farmers must make changes to their management practices to ensure that the products they are producing both minimize impacts on the environment and are safe for the consumer. Attractive approaches to managing infectious diseases might take advantage of the natural, innate immune system of the animal, especially its host-defense peptides.

The innate immune system is critical to the health of fishes, because the environmental medium, water, harbors a wide array of organisms, many of which may be pathogenic. Mucosal barriers, such as the skin, gill, and gut epithelia, provide a first line of defense against infection by producing and releasing components of the innate immune system, such as the host-defense peptides. Also known as antimicrobial peptides (AMPs), these host-defense peptides exhibit broad-spectrum activity against microorganisms such as bacteria, fungi, parasites, and viruses [8–10]. However, it is becoming increasingly evident that host-defense peptides also may have other functions in the health of an organism, including, but not limited to, antitumor, immunomodulatory (chemotaxis and opsonization), and antidiabetic effects [9,11]. Host-defense peptides have been identified in virtually all groups of living organisms, from bacteria to plants and animals. The ubiquity and potent activity of host-defense peptides suggest that they are critical to overall immune health.

One type of host-defense peptides are α-helical, amphipathic polypeptides [9] and a sub-group of these peptides is the piscidins which range from 18 to 46 amino acid residues in length [12–13]. Originally isolated as peptides from the striped bass (*Morone saxatilis*), white bass (*M*. *chrysops*), and their hybrid (*M*. *chrysops*, x *M*. *saxatilis*) [14–15], there is evidence that piscidins are present in a wide range of teleost fish taxa, including the families Moronidae, Sciaenidae, Siganidae, Belontidae, Cichlidae, Percichthyidae [14,16], Latidae, Sparidae [17], Sygnathidae [18], and Latridae [19]. The amino acid sequence of chrysophsins, antimicrobial peptides isolated from red sea bream (*Chrysophrys major*), a member of the family Sparidae, also are similar to the piscidins [20]. Piscidin gene transcripts have been cloned as cDNAs and characterized in striped bass and white bass [15], the hybrid striped bass [13], European seabass (*Dicentrarchus labrax*) [21], mandarin fish (*Siniperca chuatsi*) [22], Nile tilapia (*Oreochromis niloticus*) [23–24] and Atlantic cod (*Gadus morhua*) [25]. Genomic evidence suggests that the pleurocidins, found in many flatfish species, also are members of the piscidin family [22], as may be epinecidins of the grouper, *Epinephelus coioides* [26]. There also is evidence in expressed sequence tag (EST) databases (e.g., National Center for Biotechnology Information, NCBI) that piscidins may be found in the families Gasterosteidae (GenBank; EG589953) [27], Sebastidae (*Sebastes caurinus*; GenBank; GE811381, GE814249, GE814250; *Sebastes schlegeli*; GenBank; EY186247) [28], Adrianichthyidae (GenBank; DK138574.1, DK192306.1), Fundulidae (GenBank; EV452726, EV454324, EV455241, EV462742), Cyprinodontidae (GenBank; GE334746), and Anoplopomatidae (GenBank; GO619905) [29]. Piscidins have strong activity against both fish and human Gram-positive and Gram-negative bacterial pathogens [12,30] and have been localized to mast cells, the most abundant tissue granulocytes in vertebrates [30–31].

Host-defense peptides represent a promising alternative to traditional drugs because of their broad-spectrum antibacterial activities. Further research into the diversity and evolution of host-defense peptides may lead to a greater understanding of their functions, thus aiding research into potential therapeutic applications. We have previously reported the complete coding sequence and preliminary characterization of two novel piscidins from hybrid striped bass [13]. Here, we present information on other related host-defense peptides, along with a new piscidin classification scheme. We also further characterize the activities and functions of these host-defense peptides against several different strains of bacteria and a protozoan and provide a discussion on the novel aspects of diversification of the piscidin gene family members.

## Materials and Methods

North Carolina State University Institutional Animal Care and Use Committee (IACUC) approved this research (IACUC #08-118-0). We euthanised all animals using MS-222 following standard procedures.

### Experimental animals

Adult striped bass and white bass were propagated at the North Carolina State University Pamlico Aquaculture Field Laboratory (Aurora, NC). The fish were held separately in three outdoor 2400 L tanks (1.73 m diameter, 0.6 m working depth) according to standard culture procedures [32–33]. Hybrid striped bass (35–100 g body weight) were provided by Castle Hayne Fisheries, Inc. (Aurora, NC) and were held at North Carolina State University (Raleigh, NC) between 14°C and 23°C for at least 12 months in 1203 L round tanks fitted with a Triton sand filter (Pentair Aquatic Systems, Sanford, NC) and custom swirl separator and bioreactor. The fish were fed a commercial diet (5 mm Finfish Gold pellets, Zeigler Bros., Inc., Gardners, PA). Water temperature was monitored daily and ammonia, nitrite, pH, and salinity levels checked biweekly. Ten percent volume water changes were performed once daily.

### Bacterial cultures for antibacterial assays

Fish bacterial pathogens, *Streptococcus iniae* (stock 00–1425; isolated from rainbow trout, *Oncorhynchus mykiss*), *Lactococcus garvieae* (stock 00–1422; multiple antibiotic-resistant strain), *Vibrio anguillarum* (stock 91–3057; ATCC 19264), and *Aeromonas salmonicida* (stock 91–3053; ATCC® 7965™), and human bacterial pathogens, *Enterococcus faecalis* (stock 00–1403; ATCC 29212), *Staphylococcus aureus* (ATCC 29213), *Shigella flexneri* (stock 00–1402; ATCC 12022), and *Escherichia coli* (stock 00–1405; ATCC 25922), were cultured from frozen stocks stored in glycerol at -80°C. *L*. *garvieae*, *A*. *salmonicida*, *E*. *faecalis*, *S*. *aureus*, and *S*. *flexneri* were grown in Mueller Hinton Broth (MHB); the MHB was supplemented with 1% NaCl for *L*. *garvieae*. *S*. *iniae*, and *V*. *anguillarum* were grown in Tryptic Soy Broth (TSB) supplemented with 1.5% NaCl and *E*. *coli* was grown in Luria-Bertani Broth (LB). The fish and human pathogens were grown at 25°C and 37°C, respectively, while shaking at 200 rpm for 18 h except for *S*. *iniae*, which was grown for 48 h. The cultures were passaged at least three times each when in log phase to ensure that they had acclimated to culture.

### Molecular cloning of piscidins

Hybrid striped bass were carefully netted from aquaria and euthanized with buffered tricaine methansulfonate (MS-222; 100 mg/mL). Fifty to 100 mg gill samples were briefly rinsed with deionized water to remove any surface debris. The samples were frozen in liquid nitrogen and then stored at -80°C until nucleic acid extraction was performed. Total RNA and genomic DNA were extracted using Trizol Reagent (Invitrogen, Carlsbad, CA) and quantified by Nanodrop spectrophotometry (Thermo Fisher Scientific, Wilmington, DE).

A partial coding sequence of piscidin 3 was obtained by amplification of genomic DNA from hybrid striped bass using primers designed from European seabass expressed sequence tag (EST) sequences. One seabass EST encoding a polypeptide of high similarity to piscidin 3 was identified following a TBLASTN search [34] using the hybrid striped bass piscidin 3 polypeptide sequence [30] as the reference sequence [GenBank: FM023299 (E-value = 6e-04)]. The P3 2 F and P3 2 R1 PCR primers were designed using this EST (see S1 Table for all PCR primer sequences used in this study). Using hybrid striped bass genomic DNA extracted from gill as the template, PCR amplification was performed with these primers on a Bio-Rad iCycler thermalcycler (Bio-Rad Laboratories, Hercules, CA). The cycling protocol was: 1 cycle, 95°C for 1 min; 30 cycles, 94°C for 30 s, 52.2°C for 30 s, and 68°C for 1 min; final extension, 68°C for 5 min.

A partial coding sequence of white bass piscidin 4 was obtained during amplification of genomic DNA from hybrid striped bass using primers designed from European seabass sequences while determining the gene sequence for piscidin 4. In December 2009, four seabass ESTs encoding polypeptides of high similarity to piscidin 4 were identified following a TBLASTN search [34] using the hybrid striped bass piscidin 4 polypeptide [12] as the reference sequence [GenBank; FM019965 (E-value = 9e-11), FM025254 (E-value = 1e-10), FM019301 (E-value = 7e-07), and FM022266 (E-value = 3e-05)]. P4 5 F and P4 5 R PCR primers were designed using this EST (S1 Table). Using hybrid striped bass genomic DNA extracted from gill as the template, PCR amplification was performed with these primers on a Hybaid Px2 Thermal Cycler (Thermo Fisher Scientific, Inc., Waltham, MA, USA). The cycling protocol was: 1 cycle, 95°C for 1 min; 30 cycles, 94°C for 30 s, 52.2°C for 30 s, and 68°C for 1 min; final extension, 68°C for 5 min.

A novel 6.3 kDa antibacterial peptide corresponding to piscidins 6 and 7 was previously purified from hybrid striped bass gill and the amino acid sequence was determined by automated Edman chemical degradation (U. Silphaduang and E. J. Noga, *unpublished data*). Degenerate PCR primers HSB63 F4, HSB63 R1, R63 F3, R63 F7, and R63 R5 were designed from this peptide sequence and an EST from the rockfish, *Sebastes caurinus*, [NCBI Accession No. GE811381 (E-value = 0.31), August 2010] (S1 Table). Using hybrid striped bass genomic DNA extracted from gill as the template, PCR amplification was performed with these primers on a Bio-Rad iCycler thermalcycler (Bio-Rad Laboratories, Hercules, CA, USA). The cycling protocol was: 1 cycle, 95°C for 1 min; 30 cycles, 94°C for 30 s, 52°C for 30 s, and 68°C for 1 min; final extension, 68°C for 5 min.

Amplified PCR products corresponding to the gene sequences encoding piscidin 3, white bass piscidin 4, and piscidins 6 and 7 were electrophoresed (3% agarose), and all bands were excised and purified using GeneClean II (MP Biomedicals, Solon, OH) according to the kit protocol. Purified amplicons were cloned into a pCR 2.1-TOPO vector and transformed into chemically competent TOP10 *E*. *coli* using a TOPO TA Cloning kit (Invitrogen) and protocol. Transformant colonies were selectively grown (LB with 50 mg/mL ampicillin) for plasmid extraction (PureLink Quick Plasmid Miniprep Kit; Invitrogen) and sequencing at the University of Chicago Cancer Research Center DNA Sequencing Facility (Chicago, IL). Forward Sanger sequencing was performed on an Applied Biosystems 3730XL DNA sequencer using the M13 universal forward primer. All resulting partial cDNA sequences were aligned using the ClustalW algorithm [35] and manually assembled into contigs based on the overlapping regions.

Rapid Amplification of cDNA Ends (RACE) was used to clone the full-length gene transcript sequences encoding several piscidin forms from hybrid striped bass cDNA. Primers were designed using the exonic regions in the genomic sequences from hybrid striped bass (S1 Table): white bass and striped bass forms of piscidin 3 (RP3 F1 nest and RP3 R1 nest), the white bass form of piscidin 4 (RP4 F1, RP4 R1, RP4 F5, and RP4 R4), the white bass and striped bass forms of piscidin 6, and the striped bass form of piscidin 7 (RP63 F4, RP63 R4, and RP63 R6). Both 5’ and 3’ RACE were conducted using the FirstChoice RLM-RACE kit (Ambion, Austin, TX, USA) with total RNA from hybrid striped bass gill as the template. Primary and nested RACE-PCR amplification was performed using a Bio-Rad iCycler thermalcycler (Bio-Rad Laboratories) and the gene specific and kit-provided primers listed in S1 Table. The PCR cycling procedure was: 1 cycle, 94°C for 3 min; 35 cycles, 94°C for 30 s, 53°C (piscidin 3) or 52.2°C (55 AA peptides) for 30 s, and 72°C for 1 min; final extension, 72°C for 7 min. Cloning and sequencing of the RACE-PCR products were performed as described above.

Because the 5’ and 3’ RACE-PCR partial products for piscidins 6 and 7 were not contiguous, primers located in the 5’ and 3’ untranslated regions of the known sequence (RP63 F3, RP63 F7, and RP63 R5; S1 Table) were used to amplify contiguous sequences to create full-length gene transcripts. The PCR cycling procedure was: 1 cycle, 94°C for 3 min; 35 cycles, 94°C for 30 s, 55°C for 30 s, and 72°C for 1 min; final extension, 72°C for 7 min. Cloning and sequencing of the PCR products were performed as described above. The sequences obtained were aligned with those from the RACE-PCR to verify complete coverage of the predicted full-length gene transcripts.

### Analysis of piscidin sequences

Resulting partial cDNA sequences were aligned with ClustalW [35] and manually assembled into contigs based on the overlapping regions. Comparisons to previously reported sequences were performed by BLASTN analysis of the NCBI [36]. Alignments of the complete striped bass and white bass piscidin coding nucleotide (encoding the pre-propeptide) or polypeptide sequences deduced from the cDNAs were manually conducted using MacVector (Oxford Molecular Ltd., Cary, NC) software with the ClustalW (v 1.83) algorithm [35] and shown as sequence alignments. Multiple alignment parameters were as follows: open gap penalty = 10.0; extended gap penalty = 0.2; delay divergent = 30%; gap distance = 4; similarity matrix = gonnet. A ClustalW-formatted dendrogram also was created using the nucleotide sequences encoding the pre-propeptide of the striped bass and white bass piscidins and the nucleotide sequences encoding the hepcidins of *Larimichthys crocea* (GenBank; EU443735) and *Monopterus albus* (GenBank; GU997139). Multiple alignment parameters were as follows: open gap penalty = 10.0; extended gap penalty = 0.2; delay divergent = 30%; gap distance = 4; similarity matrix = gonnet.

The putative signal peptides were predicted using the SignalP 3.0 Server (Center for Biological Sequence Analysis, Lyngby, Denmark) [37]. The theoretical isoelectric points and predicted molecular weights were determined using the ExPASY ProtParam tool [38]. Secondary structure for the deduced peptide sequences was predicted using the Garnier procedure with GOR4 software [39]. The helical wheel diagrams were produced using EMBOSS pepwheel (European Bioinformatics Institute, Cambridge, UK) [40].

### Assignment of *Morone* piscidin gene orthology

Genomic DNA was extracted from the whole blood of striped bass, white bass, and hybrid striped bass using a Qiagen DNeasy Blood & Tissue Kit (Qiagen, Inc., Valencia, CA). Primers designed previously for cloning and Real-Time quantitative PCR (qPCR) were used to amplify the specific piscidins (1, 3, 4, 5, 6, and 7) from the genome of each species (S1 Table). PCR amplification was performed with these primers on a Bio-Rad iCycler thermalcycler (Bio-Rad Laboratories, Hercules, CA). The cycling protocol was: 1 cycle, 95°C for 1 min; 30 cycles, 94°C for 30 s, 52.2°C for 30 s, and 68°C for 1 min; final extension, 68°C for 5 min. Molecular cloning and sequencing was performed as described above. Resulting gDNA sequences were aligned using the ClustalW algorithm [35] and manually assembled based on the overlapping regions.

### Quantification of piscidin mRNA expression

Piscidin mRNA gene expression was quantified using qPCR performed as previously described with a few exceptions [41]. Briefly, total RNA was isolated from gill, foregut region of the intestine, liver, spleen, and head kidney from hybrid striped bass (N = 10 biological replicates) using Trizol reagent (Invitrogen, Carlsbad, CA). One microgram of the total RNA was used to synthesize cDNA using a High Capacity cDNA Synthesis kit (Applied Biosystems, Carlsbad, CA) following treatment with Turbo DNA-free (Ambion, Foster City, CA). RNA was quantified and checked for quality at each step using a Nanodrop ND-1000 spectrophotometer (Thermo Scientific, Wilmington, DE) and agarose gel electrophoresis, respectively. Gene expression (mRNA) of the different piscidin forms was measured in the hybrid striped bass cDNA using SYBR Green (white bass piscidins 1 and 7) or TaqMan (striped bass piscidins 1, 4, 6 and white bass piscidins 4, 5, 6) chemistries. Piscidin gene-specific primers (Invitrogen) and probes (Sigma-Aldrich, Co., St. Louis, MO) for TaqMan chemistries were designed using Beacon Designer (PREMIER Biosoft, Int., Palo Alto, CA; S1 Table). To optimize discrimination of our targets, we used fluorescent LNA (Locked Nucleic Acid) probes. LNA chemistry has the advantage over TaqMan probes by locking specific nucleic acid analogs into a rigid conformation, which increases thermal stability and improves specificity of probe hybridization. Specificity of the primers for each piscidin were verified by both forward and reverse direct sequencing of PCR products using hybrid striped bass gill and intestine cDNA as template. Optimization for appropriate annealing temperature, primer and probe concentrations, and cycling parameters was performed using pooled cDNA from the above reverse transcription reactions. One hundred ng of starting total RNA was used for qPCR analysis with Brilliant II QPCR Master Mix (Agilent Technologies, Inc., Clara, CA) containing 3 μM gene-specific primers and 2.5 μM probe. No template controls and no reverse transcription controls were incorporated into the assay. Sequenced bacterial clones were used to produce the plasmid DNA for creating the standard curves. All qPCR assays were run in triplicate wells (N = 3 technical replicates) on a 7300 Real Time PCR System (Applied Biosystems). Cycling conditions were: 1 cycle, 50°C for 2 min; One cycle, 95°C for 10 min; 40 cycles, 95°C for 15 s, 60°C for 1 min. Melting curve analysis was performed on the SYBR Green expression assays to determine primer specificity. All qPCR products were electrophoresed on agarose gels and sequenced to verify that each primer set specifically amplified a single target piscidin. Data was normalized to the starting total RNA concentration as we have previously described [42]. Pooled total RNA samples from hybrid striped bass not used in these experiments were included on each plate to normalize the data between plates. Quantification of gene expression (piscidin gene copy number/ng total RNA) was carried out by dividing the anti-log of the predicted gene copy number (predicted by comparing the mean cycle threshold (C_t_) to the serially diluted plasmid DNA standard curve) by the normalized total RNA concentrations used in the qPCR reactions.

### Piscidin peptide synthesis

The putative mature peptide region of the piscidin peptides were synthesized at Yale University School of Medicine Small Scale Peptide Synthesis (New Haven, CT) using Fmoc peptide synthesis on a Rainin Symphony instrument that provides on instrument cleavage of the peptide from the resin. Each piscidin peptide was synthesized based on the encoded amino acid sequence predicted from the cloned DNA or cDNA as follows: striped bass piscidins 1, 4, and 7 and white bass piscidins 1, 5, and 6. The striped bass and white bass piscidin 3 peptide sequences are identical and therefore this synthetic peptide represented the activity of the protein expressed in either species. The air-dried peptides were reconstituted in 0.01% acetic acid solution to a starting concentration of 4 mg/mL for all antibacterial and antiprotozoal assays.

### Enzyme-Linked Immnuosorbent Assay (ELISA) for striped bass piscidin 4

The ELISA for striped bass piscidin 4 was performed as previously reported [17]. Fifty μL (weight/volume) of gill and intestinal tissues were removed from the same fish used in the gene expression analysis. The samples were boiled for 5 min in 1% acetic acid and immediately stored on wet ice. The samples were then stored long term at -80°C until protein extraction was performed. Extraction was done by homogenizing the tissue for 30 s on ice with a Polytron PT1200 tissue homogenizer (Kinematica, Inc.) and centrifuging at 12,000 x g for 15 min at 4°C. The supernatant was removed and stored at -80°C until the assay was performed. Samples for sandwich ELISA analysis were analyzed in duplicate (N = 2 technical replicates). Striped bass piscidin 4 concentrations were calculated by comparing absorbance at A_260_ to a standard curve generated using pure synthetic piscidin 4. Extracts of tissues that had the highest and lowest expression of piscidin 4 were pooled together to produce a representative sample that was used as a control for the assay.

### Microbroth dilution assay

The minimum inhibitory concentrations (MIC) and minimum bactericidal concentrations (MBC) were determined as previously described [12,30] following standard methods recommended by the National Committee of Laboratory Safety and Standards for MIC_90_ and MBC determinations of clinical isolates with some modifications. Overnight bacterial cultures were diluted to 80–88% transmittance in their appropriate medium. The cultures were then diluted again to 1:200 and 100 μL was pipetted into each of 3 wells in 11 columns of a 96-well plate (Corning, Inc., Tewksbury, MA), the 12^th^ column loaded with media without bacteria as a sterility control and blank.

The synthetic piscidin peptides (piscidins 1, 4, and 7 from striped bass; piscidins 1, 5, and 6 from white bass; and piscidin 3 corresponding to both striped bass and white bass) were prepared by diluting the 4 mg/mL stocks to 2 mg/mL in 0.2% bovine serum albumin (BSA) + 0.01% acetic acid. These were then serially diluted to a concentration of 3.9 μg/mL in the same diluent. The piscidin dilutions were added to the first 10 columns of the 96-well plate containing bacteria, column 10 was loaded with diluent only to act as a positive growth control. The final concentrations tested were 200 μg/mL to 0.39 μg/mL. The plates were incubated with shaking at 200 rpm for 18 h, except for *S*. *iniae*, which was incubated for 48 h. Fish pathogens and human pathogens were incubated at 25°C and 37°C, respectively. Once visible growth was observed in the controls, the plates were read at OD_630_ on a BioTek EL800 plate reader (BioTek, Winooski, VT). The MIC was taken as the lowest concentration of piscidin that reduced growth when compared to the growth control by more than 50%. To determine the MBC, 20 μL of the media from the wells of the three lowest piscidin concentrations that had no visible growth of bacteria were plated onto the appropriate media plates and incubated as described above. Colonies were counted to determine whether growth was equal to or less than 0.1% of the colony forming units (CFUs) in the original inoculum. The MBC was defined as the lowest concentration of piscidin that killed at least 99.9% of the CFUs in the original inoculum.

### Antiparasitic activity assay

An anexic culture of *Tetrahymena pyriformis* (#13–1620; Carolina Biological Supply, Burlington, NC) was maintained in *Tetrahymena* medium (Carolina Biological Supply) and incubated aerobically at 25°C. Approximately 100–150 mL of culture was aseptically pipetted into 5 mL of fresh medium every 4 days. Prior to use in antiparasitic assays, cultures were diluted 1:20 with sterile water. After gentle mixing, the suspension was counted and adjusted to a final concentration of 20–25 ciliates per 10 mL.

The PC_min_ (minimum parasiticidal concentration, the lowest concentration of synthetic piscidins that killed at least one *T*. *pyriformis*) and the PC_100_ (100% parasiticidal concentration, the lowest concentration of piscidin that killed all *T*. *pyriformis*) were determined as described previously [43]. All experiments were performed at 25°C. Briefly, the synthetic peptides (piscidins 4 and 7 from striped bass; piscidins 5 and 6 from white bass) were serially diluted in 0.01% HAc/0.2% BSA at 10x their test concentration (peptides were serially diluted from 80 mM to 1.25 mM). Ten μL of each dilution were added to replicate wells of a flat-bottom 96-well plate (Becton Dickinson) containing 80 mL of endotoxin-free water. As a control, 10 mL of the above diluent was added to 80 mL of endotoxin-free water in replicate wells. Ten μL of the *T*. *pyriformis* suspension above were added to each of the replicate wells starting with the lowest concentration followed by observations for at least 15 min before adding the suspension to the next highest concentration. Observations were made using Nikon Diaphot inverted microscope (Nikon Instruments, USA) every 5 min during the first 30 min and then every 15 min during the next 60 min to identify lysis or loss of mobility of cells. Observations also were made at 3 h post-addition and then again at 24 h. These results were compared to those previously observed [43–44]. Digital images of the effects of the piscidins on the protozoans also were recorded.

### Statistical analysis

All statistical analyses were performed using JMP 10 (SAS Institute, Inc., Cary, NC). Comparisons of means were made using Tukey-Kramer Honestly Significant Difference (HSD) tests. Significance was set at a nominal *P* value cutoff of 0.05 (a priori α = 0.05).

## Results

### Piscidin 3

**Fig 1:**
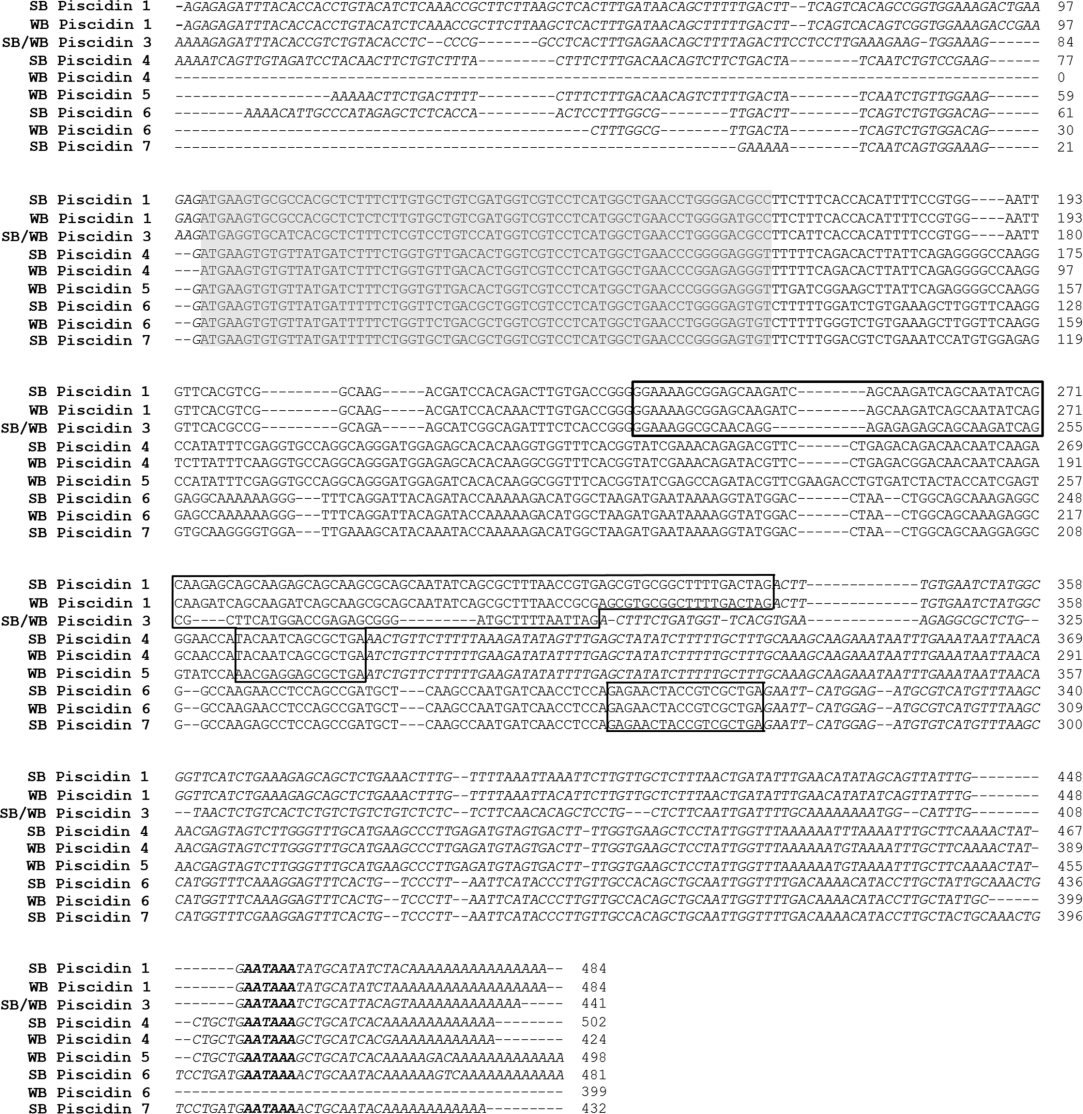
Nucleotide sequences for hybrid striped bass piscidin gene transcripts. The piscidin labels indicate from which parent species that particular piscidin originates (SB, striped bass; WB, white bass). The predicted 22 amino acid signal peptide, shown by the gray shading, was similar in all piscidins. The prodomains are in boxes. Both the 5’ and 3’ untranslated regions are shown in italics. The polyadenylation signals are bolded. The cDNA sequence for both striped bass and white bass piscidin 3 are the same, as indicated by the label.

**Fig 2:**
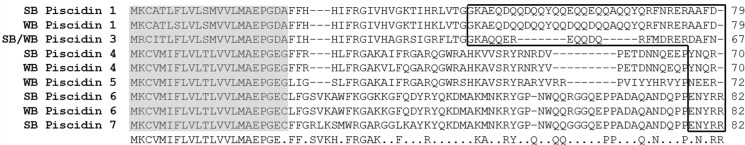
Predicted amino acid sequences for piscidins described in striped bass, white bass, and their hybrid. The predicted 22 amino acid signal peptide, shown by the gray shading, was similar in all piscidins. The prodomains are in boxes and exhibit considerable variation between piscidin forms. The consensus sequence is shown below the alignment. The translated peptide sequence for both striped bass and white bass piscidin 3 are the same, as indicated by the label.

The partial sequences obtained from 5’ and 3’ RACE-PCR of hybrid striped bass cDNA using primers specific to piscidin 3 were aligned and together formed a single contiguous 441 bp transcript sequence (Fig 1) [GenBank; KX231319 (striped bass), KX231323 (white bass)]. This piscidin 3 gene transcript included 5’ and 3’ untranslated regions (UTRs) and encoded a conserved putative 22 amino acid (AA) signal peptide, 22 AA mature peptide, and 23 AA prodomain (Fig 2). The 3’ UTR included a poly(A) signal prior to the polyadenylation site.

Primers specific for piscidin 3 amplified two distinct products (663 bp and 723 bp in length) from hybrid striped bass genomic DNA. The larger of these products also was uniquely observed in the genomic DNA of striped bass (GenBank; KX231314),whereas the smaller product was present in white bass (GenBank; KX231310) (S1 Fig), indicating that piscidin 3 has distinct orthologues in the striped bass and white bass and that both of these forms are present in the hybrid striped bass genome. Each of these products included a region of two exons separated by an intron. The exonic regions of the piscidin 3 loci were identical between the striped bass and the white bass and both correspond to the 441 bp gene transcript, indicating that the piscidin 3 exons encode a peptide sequence that is identical in striped bass and white bass. In contrast, the intron contains an imperfect microsatellite short tandem repeat and the alleles at this locus vary in size between the striped bass and white bass. The repeats of the smaller hybrid striped bass product and the white bass product were the same [(TC)_4_(TG)_12_(AGTG)_3_(TG)_2_]. Those of the larger hybrid striped bass product [(TG)_45_(AGTG)_2_(TG)_3_] and the striped bass product [(TG)_34_(AGTG)_2_(TG)_3_] shared similar repeat structure, but differed in the number of 5’ TG repeats (S1 Fig).

### Piscidins 4 and 5

**Fig 3:**
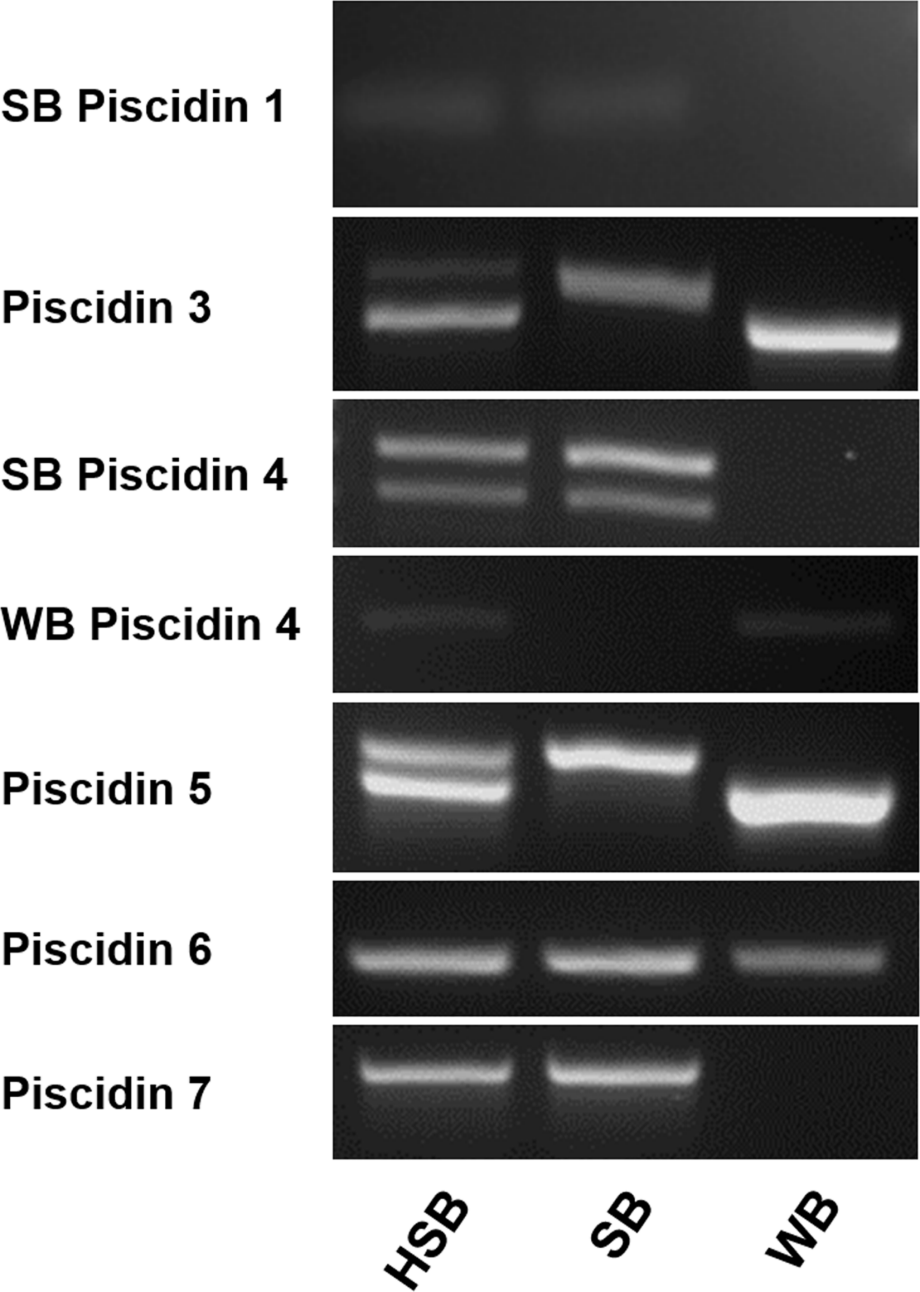
Agarose gel electrophoresis using genomic DNA as template along with piscidin gene specific primers. Bands in each of the parent species can be compared with those of the HSB. WB Piscidin 1 was not included because the primers designed crossed exon-exon boundries. SB = striped bass, WB = white bass, and HSB = hybrid striped bass.

The sequences of striped bass piscidins 4 and 5 have already been described (Salger et al. 2011). Here, a 424 bp long gene transcript sequence orthologous to piscidin 4 was cloned from white bass cDNA (GenBank; KX231324) (Fig 1). The sequence contained a start codon and encoded a conserved putative 22 AA signal peptide, 44 AA mature peptide, and 4 AA prodomain. The deduced peptide sequence shared 91.4% and 77.8% identity with piscidins 4 and 5, respectively from striped bass (Table 1) indicating that piscidin 4 has distinct orthologues in the striped bass and white bass and that both forms are expressed in the hybrid striped bass. This is further supported by the genomic DNA sequence evidence of piscidin 4 in striped bass, white bass, and their hybrid (GenBank; KX231315 and GenBank; KX231311, respectively) (Fig 3). Although a 5’ UTR was not observed, a complete 3’ UTR was identified including the poly(A) signal in the piscidin 4 gene transcripts. White bass and striped bass piscidin 4 share similar predicted mature peptide secondary structures, with the first 25 AAs forming a coil-helix and the last 19 AAs forming a coil-β-sheet. While striped bass piscidin 5 shares a similar predicted structure with the first 25 AAs of piscidin 4, its last 21 AAs form a predicted helix-coil-β-sheet, different from that of piscidin 4 in both species.

**Table 1:** Similarity and identity scores based on the deduced peptides of all currently known hybrid striped bass piscidins, *Larimichthys crocea* hepcidin (Lc Hepcidin; GenBank assession EU443735), and *Monopterus albus* hepcidin (Ma Hepcidin; GenBank assession GU997139).

	*Similarity Scores (%)*										
	Lc Hep	Ma Hep	WB P1	WB P5	WB P4	WB P6	SB P1	SB P4	SB P6	SB P7	P3
**Lc Hepcidin**	100	47.8	9.3	15.1	12.8	12.5	9.3	12.8	12.5	10.2	7.0
**Ma Hepcidin**	62.2	100	7.8	12.2	13.3	9.8	7.8	12.2	8.7	10.9	4.4
**WB Piscidin 1**	27.9	27.8	100	25.3	29.1	25.3	94.9	27.8	25.3	27.7	58.2
**WB Piscidin 5**	20.9	22.2	40.5	100	69.4	42.7	26.6	70.8	41.5	37.8	27.6
**WB Piscidin 4**	24.4	27.8	48.1	77.8	100	43.9	30.4	91.4	42.7	40.2	28.0
**WB Piscidin 6**	22.7	21.7	42.2	48.8	48.8	100	26.5	42.7	98.8	82.9	22.0
**SB Piscidin 1**	26.7	26.7	98.7	41.8	49.4	44.6	100	29.1	26.5	28.9	60.8
**SB Piscidin 4**	24.4	27.8	45.6	76.4	95.7	50.0	46.8	100	41.5	40.2	29.3
**SB Piscidin 6**	22.7	20.7	42.2	47.6	47.6	98.8	44.6	48.8	100	81.7	22.0
**SB Piscidin 7**	20.5	23.9	43.4	45.1	48.8	90.2	45.8	50.0	89.0	100	23.2
**Piscidin 3**	20.9	20.0	70.9	39.5	42.7	42.7	72.2	41.3	42.7	43.9	100
***Identity Scores (%)***											

SB indicates striped bass and WB indicates white bass. The translated peptide sequence for both striped bass and white bass piscidin 3 are the same.

Two products corresponding to piscidin 5 were amplified from genomic DNA of hybrid striped bass (Fig 3). The smaller of these products was uniquely observed in the genomic DNA of white bass (GenBank; KX231312) and the larger present in striped bass (Genbank; KX232424) (Fig 3) indicating that two distinct orthologues of piscidin 5 are present in the hybrid striped bass genome. The open reading frame of the smaller piscidin 5 gene in hybrid striped bass and white bass corresponds to the previously published piscidin 5 gene transcript sequence [13], whereas the larger hybrid striped bass and striped bass piscidin 5 gene codes for an incomplete peptide (S2 Fig).

The encoded polypeptide sequence of striped bass piscidin 5 is truncated in comparison to the white bass form (S2 Fig) at the amino-terminal end presumably due to loss of an exon containing the start codon. This is further supported by observation that a region corresponding to the 5’ end of the piscidin 5 gene transcript could not be amplified from striped bass or hybrid striped bass genomic DNA although other regions of the gene were amplified, indicating that this 5’ sequence is either missing or grossly different from the white bass form. Additionally, several unsuccessful attempts were made to clone the gene transcript from the cDNA of various striped bass and hybrid striped bass tissues, indicating that the gene is likely not expressed as a functional protein. In any case, it appears that the piscidin 5 has been lost in the striped bass and retained in the white bass and that the hybrid striped bass only expresses the single white bass piscidin 5 form (Fig 3).

### Piscidins 6 and 7

Degenerate primers designed to amplify a partial gene sequence corresponding to a novel 6.3 kDa antibacterial peptide purified from hybrid striped bass gill generated a 340 bp sequence from hybrid striped bass genomic DNA (GenBank; KX232425), which included two exonic regions that together encode an 11 AA long partial protein corresponding to AAs 14–24 of the purified peptide. The RACE-PCR sequences of hybrid striped bass were then aligned to the protein coding regions in this 340 bp genomic DNA fragment and three similar contiguous gene transcript sequences of 481 bp (GenBank; KX231321), 399 bp (GenBank; KX231326), and 432 bp (GenBank; KX231322) were discovered (Fig 1). Amplification of the entire gene transcript from cDNA verified that these genomic sequences corresponded (at least in part) to the transcribed regions of the genes.

A BLASTp search using the derived prepropeptides as query sequences assigns the first 27 AAs of each of these three gene transcripts to the Antimicrobial 12 superfamily, of which pleurocidins are representative. These deduced peptides shared a similar basic pre-propeptide structure as all other piscidins: a 22 AA conserved signal peptide, a 55 AA mature peptide, and a 5 AA prodomain (Fig 2). Specific primers designed to amplify a 254 bp long product corresponding to the 481 bp and 399 bp gene transcript sequences resulted in two distinct amplicons from hybrid striped bass genomic DNA, one product each derived from striped bass and white bass (GenBank; KX231316 and GenBank; KX231313, respectively) (Fig 3). These products correspond to striped bass and white bass piscidin 6 forms. Specific primers designed to amplify the 432 bp gene transcript sequence amplified a single product from hybrid striped bass and striped bass genomic DNA corresponding to piscidin 7 (GenBank; KX231318 and GenBank; KX231317, respectively) (Fig 3). A piscidin 7 orthologue was not detected in white bass despite several attempts at PCR using various combinations of different forward and reverse primers.

**Fig 4:**
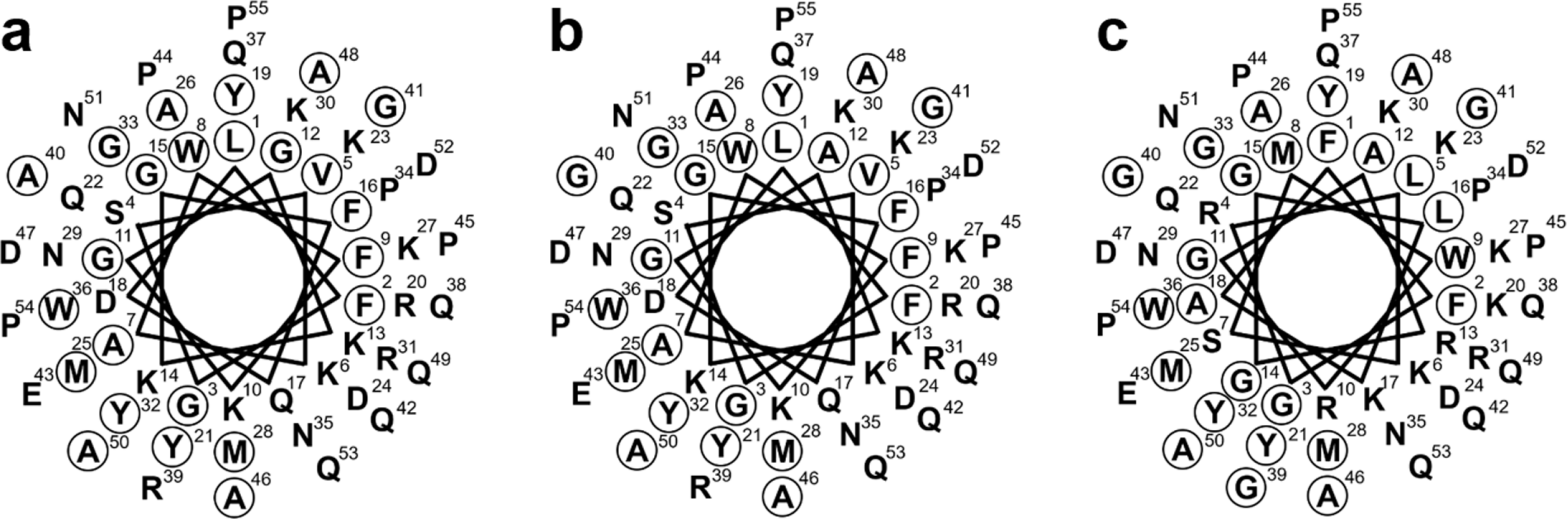
Helical wheel diagrams of piscidins 6 and 7. (a) Striped bass piscidin 6. (b) White bass piscidin 6. (c) Striped bass piscidin 7. An amphipathic nature is suggested in all three piscidins by the alignment of the hydrophobic residues (circled amino acids) along one side of the helix and the hydrophilic residues along the other.

The α-helical plots of the three mature piscidin 6 and 7 peptides were performed using EMBOSS pepwheel software. In all, the α-helical plots suggest that these three piscidins are all amphipathic in nature (Fig 4), again supporting the conclusion that these are a new class of piscidins found in the hybrid striped bass.

**Fig 5:**
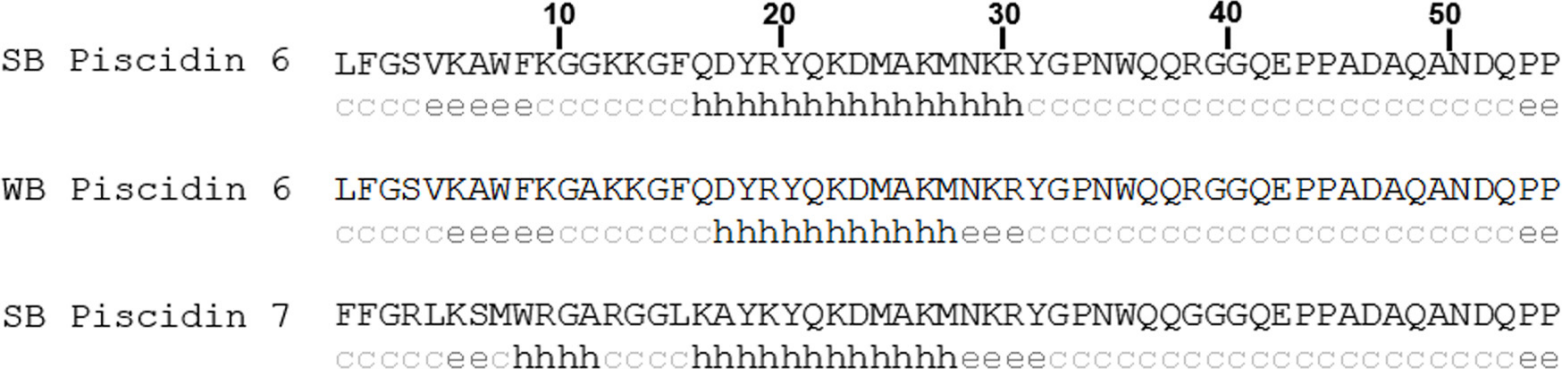
The predicted secondary structure of the mature peptides for the Class III piscidins. The secondary structure of the amino-terminal end of Class III piscidins consists of a coil-β-sheet-coil-helix structure, which is different from those of Class I and Class II piscidins. c = coil, h = helix, e = sheet.

The predicted secondary structure of the mature piscidin 6 and 7 peptides differs from those of the other piscidins in that the N-terminal region follows a coil-β-sheet-coil-helix structure (Fig 5). Additionally, the mature peptide is longer (55 AA) than other previously described piscidin isoforms (22 AA and 44–46 AA). The encoded piscidin 6 and 7 peptides are predicted to have molecular weight of approximately 6,229 Da and 6,318 Da, respectively, in close agreement to the size of the antibacterial peptide purified from hybrid striped bass gill.

### Proposed reclassification of piscidins

Based on the evidence provided here along with that of Lauth et al. (2002), piscidins 1 and 2 are orthologues and will herein be named as such: piscidin 1 being striped bass piscidin 1 and piscidin 2 being the white bass piscidin 1 orthologue. Other piscidins were named 3–7 in order of their discovery and the striped bass and white bass orthologues were identified in the hybrid striped bass as described above (see Table 2). Piscidins 3, 4, and 6 have orthologues in both the striped bass and white bass and both orthologues are present in the genomic DNA of hybrid striped bass as would be anticipated. Piscidin 5 is present in the genomic DNA of white bass, striped bass, and their hybrid, however only the white bass orthologue appears to be functionally expressed as a gene transcript; the striped bass piscidin 5 appears to be a pseudogene. Piscidin 7 is only detected in the genome sequence of striped bass and the hybrid striped bass and it also is expressed in both of these fish. The white bass does not appear to have a piscidin 7 isoform and the locus (or remnant thereof) could not be detected by PCR.

**Table 2:** Lines of evidential inquiry of different forms of piscidins in hybrid striped bass, striped bass, and white bass.

	Hybrid Striped Bass	Striped Bass	White Bass
**Piscidin 1**	G, E	G, E	G, E
**Piscidin 3**	G, E	G	G
**Piscidin 4**	G, E	G	G
**Piscidin 5**	G, E	G, not expressed	G
**Piscidin 6**	G, E	G	G
**Piscidin 7**	G, E	G	NULL

G = genomic evidence, E = gene expression evidence, and NULL = not present/detected.

**Fig 6:**
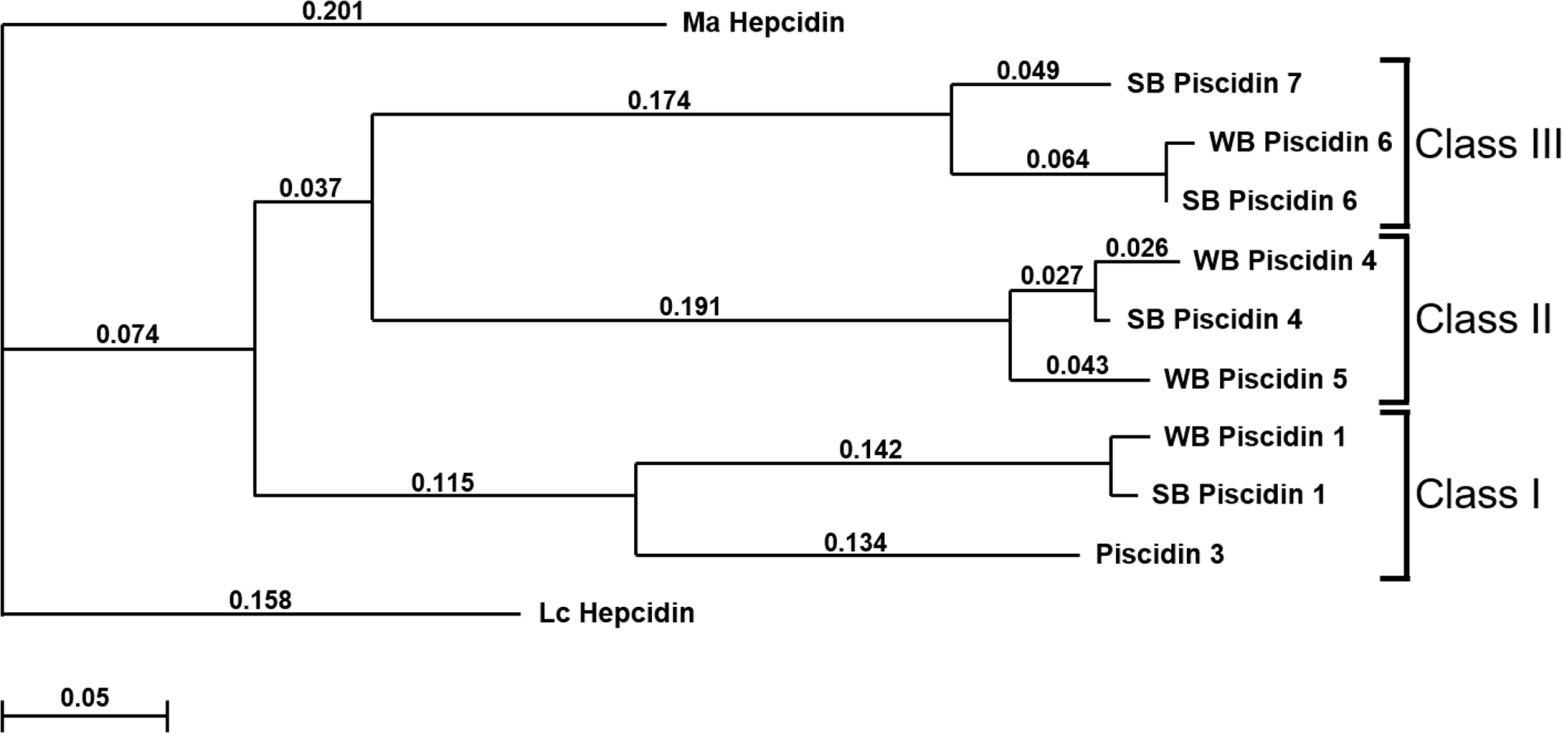
ClustalW-formatted dendrogram showing relationships among piscidins based on multiple alignments of the nucleotide sequence encoding the pre-propeptide. The piscidins are grouped into three classes (Class I, Class II, and Class III). Alignments were rooted to *Larimichthys crocea* hepcidin (Lc Hepcidin; GenBank accession EU443735) and *Monopterus albus* hepcidin (Ma Hepcidin; GenBank accession GU997139) protein coding sequences. Open gap penalty = 10.0; extended gap penalty = 0.2; delay divergent = 30%; gap distance = 4; similarity matrix = gonnet; SB = striped bass; WB = white bass. Numbers above each of the branches represent *p*-distances.

These different piscidin isoforms can be divided into three separate classes based on our current understanding of their structures: Class I representing the 22 AA piscidins 1 and 3, Class II representing the 44–46 AA piscidins 4 and 5, and Class III representing the 55 AA piscidins 6 and 7. This also is supported by the ClustalW dendrogram of piscidin gene coding sequences (Fig 6).

### Tissue distribution of piscidin gene transcript expression

**Fig 7:**
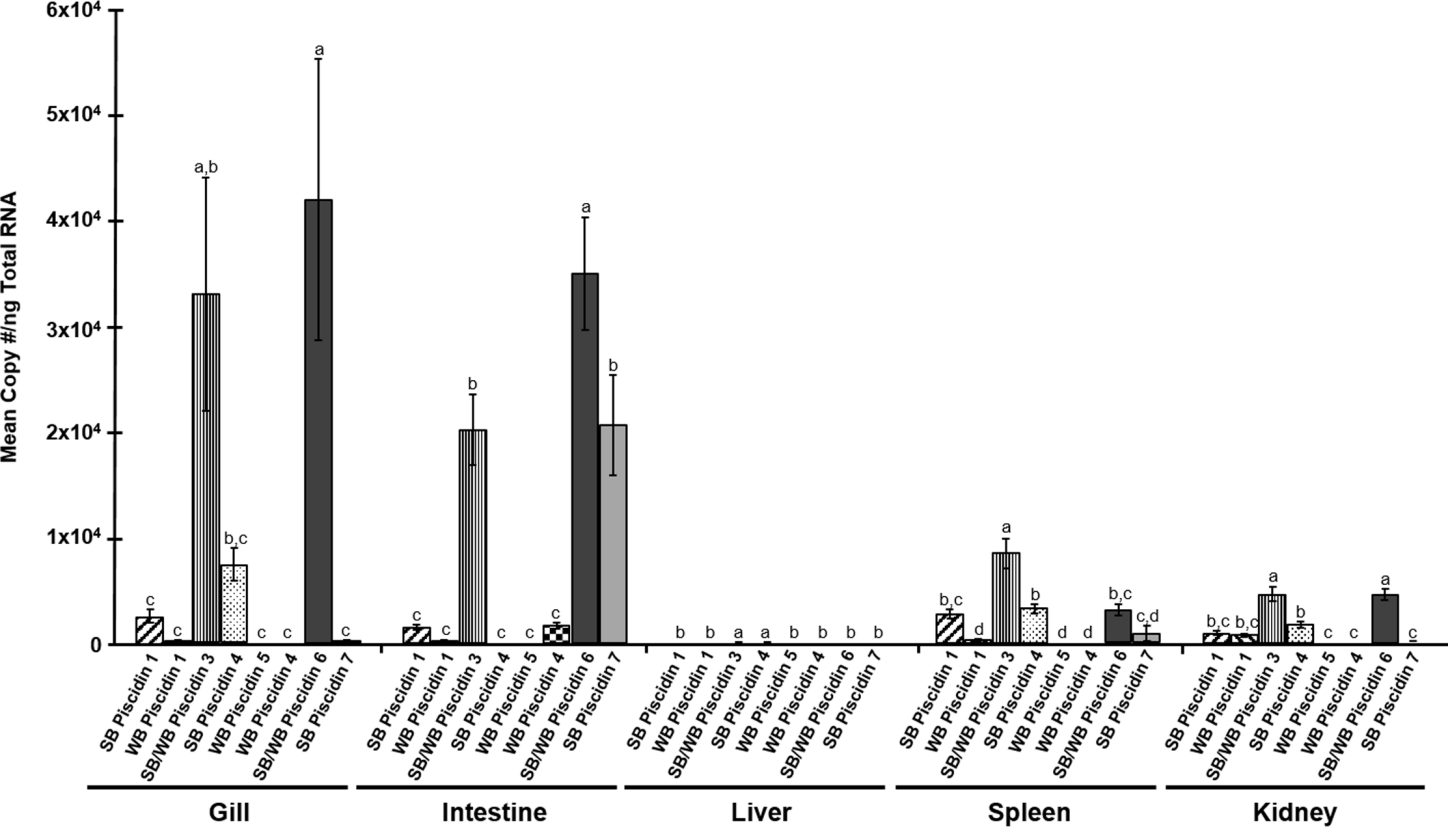
Constitutive tissue expression of piscidin gene transcripts in different tissues of hybrid striped bass. Bars indicate mean copy number per ng Total RNA ± SE. The letters above each bar denote statistical differences between the piscidins within each tissue (Tukey’s HSD; *P* = 0.05). Primers designed for piscidins 3 and 6 measured the expression of both striped bass and white bass forms in the hybrid striped bass.

Melting curve analysis indicated that there was only measurement of a single product in the SYBR Green qPCR assays. Direct sequencing of the qPCR products verified that each primer set specifically amplified the target piscidins in all qPCR assays. This, along with the TaqMan chemistry and LNA probes, provided confidence that each assay specifically quantified the genes of interest. The qPCR efficiency of the assays ranged from 95–110%. A comparison of piscidin gene expression levels in each tissue is shown in Fig 7 and S2 Table. There was very little expression in any tissue of white bass Piscidin 4 or of any piscidins in the liver.

### Striped bass piscidin 4 concentrations

The striped bass piscidin 4 peptide concentrations in the gill varied from 5 ng/mL to 2200 ng/mL with a mean ± SE of 931.6 ± 254.3 ng/mL (130.3 ng/mg tissue, 55.4 ng/mg tissue SE). The intestine extracts had little to no measurable striped bass piscidin 4 peptide at a 1:50 dilution.

### Antibacterial and antiprotozoal activity of piscidins

The activity against bacterial and protozoal pathogens was assessed using synthetic peptides of all piscidins except white bass Piscidin 4 and white bass piscidin 6. The activities against all bacterial pathogens were similar between striped bass piscidin 1 and white bass piscidin 1. Piscidin 3 (the sequence of which is identical for both striped bass and white bass) MICs and MBCs for fish pathogens were similar to striped bass and white bass piscidins 1 except for *L*. *garvieae* which was higher in piscidin 3. The MICs and MBCs for piscidin 3 were generally higher against most human bacterial pathogens than piscidin 1 of either striped bass or white bass. Striped bass piscidin 4 and white bass piscidin 5 had similar activities against all bacterial strains tested. White bass piscidin 6 and striped bass piscidin 7 had little to no antibacterial activity against any strains tested. None of the piscidins tested had activity against the *A*. *hydrophila* type strain used in these assays (Table 3).

**Table 3:** Minimum inhibitory concentrations (MIC) and minimum bactericidal concentrations (MBC) of various synthetic hybrid striped bass piscidins against fish and human bacterial pathogens.

		MIC (uM)							MBC (uM)						
	Gram	SB P1	WB P1	SB/WB P3	SB P4	WB P5	WB P6	SB P7	SB P1	WB P1	SB/WB P3	SB P4	WB P5	WB P6	SB P7
*Fish pathogens*	** **	** **	** **	** **	** **	** **	** **	** **	** **	** **	** **	** **	** **	** **	** **
*Streptococcus iniae*	+	1.22	2.46	2.51	4.7	2.26–4.52	>31.59	>32.11	1.22	2.46	2.51	4.7	4.52	>31.59	>32.11
*Lactococcus garvieae*	+	1.22	0.61	20.07	9.41	9.03	0.99	32.11	1.22	2.46	5.02	9.41	2.26	>31.59	>32.11
*Vibrio anguillarum*	-	2.43–4.86	4.91	2.51–5.02	1.18	4.52	>31.59	8.03	9.72	9.83	20.06	2.35	9.03	>31.59	>32.11
*Aeromonas salmonicida*	-	>77.76	>78.61	>80.26	>37.64	>36.12	>31.59	>32.11	>77.6	>78.61	>80.26	>37.64	>36.12	>31.59	>32.11
*Human pathogens*															
*Enterococcus faecalis*	+	1.22	1.23	1.26	1.18	4.52	>31.59	>32.11	2.43	2.46	10.03	37.64	4.52	>31.59	>32.11
*Staphylococcus aureus*	+	2.43	2.46	ND	1.18	4.52	>31.59	>32.11	2.43	2.46	ND	2.35	4.52	>31.59	>32.11
*Shigella flexneri*	-	2.43–4.86	2.46	20.07	1.18–2.35	1.13–2.26	>31.59	>32.11	4.86	2.46	40.13	1.18	2.26	>31.59	>32.11
*E*. *coli*	-	4.86	4.91	80.26	9.41	1.13	>31.59	>32.11	>77.6	>78.61	80.26	9.41	4.52	>31.59	>32.11

The striped bass (SB) and white bass (WB) piscidin 3 peptide sequence is the same in both species.

ND indicates that the activity was not determined in the assay.

**Fig 8:**
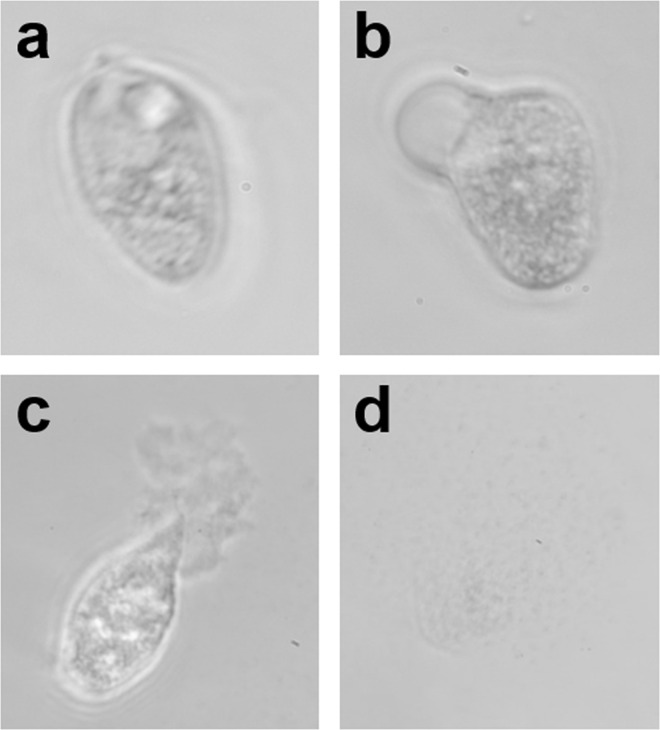
Piscidin 7 kills the ciliated protozoan *Tetrahymena pyriformis* by causing membrane disruption. (a) *T*. *pyriformis* in media with no piscidin added. Notice the oval shape and well-defined contractile vacuole at the basal end of the protozoan (top). (b) Blebbing occurs following the addition of a 12 μg/mL of striped bass piscidin 7. (c) After addition of 25 μg/mL of striped bass piscidin 7 the pellicle begins to break down and rupture. (d) There is complete dissolution of the pellicle following addition of >50 μg/mL of striped bass piscidin 7 to the culture media.

Striped bass piscidin 4, white bass piscidin 5, white bass piscidin 6, and striped bass piscidin 7 had higher antiprotozoal activity than striped bass piscidin 1 or white bass piscidin 1. The PC_100_ for striped bass piscidin 4, white bass piscidin 5, white bass piscidin 6, and striped bass piscidin 7 were similar whereas white bass piscidin 6 and striped bass piscidin 7 had higher PC_min_ activity than striped bass piscidin 4 and white bass piscidin 5 (Table 4). Images of the protozoan *T*. *pyriformis* before and after administration of piscidins are shown in Fig 8.

**Table 4:** Minimum parasiticidal concentrations (PC_min_) and 100% parasiticidal concentrations (PC_100_) of various synthetic hybrid striped bass piscidins against the ciliated protozoan, *Tetrahymena pyriformis*.

	PC_min_ (uM)						PC_100_ (uM)					
	SB P1	WB P1	SB P4	WB P5	WB P6	SB P7	SB P1	WB P1	SB P4	WB P5	WB P6	SB P7
*Tetrahymena pyriformis*	2.5	ND	0.59	0.56	<0.25	<0.25	5	2.48	1.18	1.10	0.99	1.00

The striped bass (SB) and white bass (WB) piscidins are indicated.

## Discussion

These results provide a significant expansion of the piscidin family of antimicrobial peptides from fishes of genus *Morone*, which now includes a novel, high molecular weight group with a 55 AA mature peptide. We have classified the different *Morone* piscidins as Class I (the 22 AA pisidins 1 and 3), Class II (the 44–46 AA piscidins 4 and 5), and Class III (the 55 AA piscidins 6 and 7). Based on peptide structure and size, phylogenetic analysis, gene expression, and anti-microbial activity it appears that the three different groups of piscidins may have different functions in the innate immune system.

With the data provided in the current paper, at least 9 piscidins have now been identified in striped bass and/or white bass and expression of 8 of the orthologous forms have been verified in the hybrid striped bass. The hybrid is preferred for aquaculture, because it displays greater tolerance to varying water salinities, has a better feed conversion ratio, and superior stress and disease resistance compared to either parental species [45]. Greater disease resistance may be partially due to the additive contribution of piscidins from each progenitor species, however this remains to be proven. It was previously reported that hybrid striped bass gain one putative piscidin 1 ortholog (allele) from each parent [15]. We provide more evidence in support of this observation and, with the exceptions of the striped bass piscidin 5 and white bass piscidin 7 forms, each of the functional striped bass and white bass piscidin orthologs are contributed as alleles to the hybrid striped bass genome. At present, this is at least six different piscidin loci that we are aware of; 4 loci have 2 alleles (piscidins 1, 3, 4, and 6) and 2 loci have only 1 functional allele (piscidins 5 and 7) derived from the parental species. The white bass and striped bass piscidin orthologs all differ in amino acid sequence, with the exception of piscidin 3. The piscidin 3 gene sequence differs between white bass and striped bass at an intronic microsatellite region (S1 Fig), however the encoded peptide sequence is identical between both species.

Orthologous piscidins from the striped bass and white bass share similar structures and this implies conservation of function. Piscidin structure, however, varies between the three classes, implying a diversity of function. The small Class I piscidins are the most potent antibacterial agents, but have weaker antiprotozoal activities than the larger piscidins in Class III (Tables 3 and 4). In contrast, Class III piscidins have limited to no antibacterial activity, but potent antiprotozoal activity. Class II piscidins, intermediate in size, have an intermediate range of antibacterial and antiprotozoal activities.

In order to determine the activity of piscidins against protozoa, we used the ciliated parasite, *Tetrahymena pyriformis*. Like other ciliates, it possesses a pellicle supporting the cilia and plasma membrane and, being a freshwater protozoan, has a contractile vacuole that aids in regulating the cell volume and osmoregulation [46]. The mechanism of action of most cationic antimicrobial peptides is generally thought to occur by interacting with the anionic surface components of non-self cells. At low concentrations of piscidin (3–6 μg/mL depending on piscidin tested), the contractile vacuole of *T*. *pyriformis* repetitively expanded and contracted at a rate of one cycle about every 1 to 2 minutes presumably to maintain its internal cell volume, a rate faster than that of the ciliates maintained in water without addition of any piscidins (*data not shown*). At higher piscidin concentrations (12–50 μg/mL depending on piscidin tested), damage to the pellicle was observed, along with the formation of demarcation vesicles, a process known as “blebbing” (Fig 8B). This is seen in *Paramecium* when exposed to detergents [47]. It is thought that the detergents disrupt microfilaments that are stabilized by microtubules and cause the plasma membrane to form demarcation vesicles. At even higher piscidin concentrations (>50 μg/mL), the plasma membrane begins to break down (Fig 8C and 8D). When this occurs, the protozoans are immobilized followed shortly by leakage of cytoplasm from the damaged membrane. While the precise mechanism by which piscidins function is unclear, studies do support that they all can function via membrane disruption.

White bass piscidins 4 and 5 of Class II have similar antibacterial and antiprotozoal activities (Tables 3 and 4), suggesting that the active region of the peptide is the α-helical N-terminal region that is highly conserved between the two piscidin forms (Fig 2) [13]. The N-terminal region has been shown to intercalate into the phospholipid bilayer of membranes when analyzing tryptophan quenching data from striped bass piscidin 4 [48], which also has a similar structure. This tryptophan residue is located at position 20 from the N-terminus of striped bass piscidin 4. The quenching data indicate that striped bass piscidin 4 may be embedded parallel in the microbial cytoplasmic membranes, leading it to interact with the membrane following the carpet model of membrane disruption [49–50]. Therefore, the Class II piscidins from white bass may also function in this manner, since they have similar secondary structures in this region of the peptide.

The main structural differences between piscidins within Class II occur in the C-terminal region of the peptides. The C-terminal region of piscidin 5 has a helix and coil region followed by a β-sheet at the end, whereas the C-terminal region of piscidin 4 has two sheet regions separated by a coil [13]. This β-sheet region is similar to carbohydrate and lipopolysaccharide binding motifs, which are known to be important for pattern recognition function in the innate immune system [51–53]. This variation may relate to the specificity of Class II piscidins from the striped bass and white bass to particular microorganisms.

As mentioned above, white bass have both piscidin 4 and 5 types, whereas striped bass only have the piscidin 4 type. Real time qPCR analysis shows that the piscidin 4 gene transcripts are expressed mainly in the gill, with very low expression in the foregut region of the intestine (Fig 7 and S2 Table). The protein measurements also support this for striped bass piscidin 4, as the mean concentration was high in the gill but undetectable in intestine. Piscidin 5 gene transcripts were expressed only in white bass intestine, suggesting that there is a tissue-specific profile within Class II piscidins (Fig 7 and S2 Table).

Class III piscidins also are differentially expressed in tissues of hybrid striped bass (Fig 7 and S2 Table). Piscidin 6 is expressed predominantly in gill and intestine, while piscidin 7 is expressed mainly in the intestine. Significant expression of piscidin 6 also was detected in the kidney; however, the transcript levels were lower than in gill and intestine. Both striped bass and white bass have a gene encoding piscidin 6, however only the striped bass has a gene encoding piscidin 7 and, as with piscidin 5, only one form of piscidin 7 was detected in hybrid striped bass corresponding to the striped bass sequence (Fig 3).

White bass live their entire lives in freshwater whereas striped bass are anadromous spending their adult lives in saltwater and migrating to freshwater for spawning [45]. There is evidence that the composition of microbial communities associated with fishes is influenced by many environmental factors, most significantly by salinity [54–56]. Alterations in microbial flora during sea water adaptation occur as salinity-tolerant microbes proliferate while other less tolerant organisms do not [54]. The differences in microbial communities between fresh and salt water may relate to the reasons why white bass and striped bass have unique piscidin 5 and piscidin 7 forms, respectively (Fig 3). We have shown that piscidin 7 has antiprotozoal properties whereas piscidin 5 has a balance of antibacterial and antiprotozoal properties (Tables 3 and 4). Bacterial diversity is greater in freshwater sediments than in either intertidal or marine sediments [57–59], which may indicate a propensity of the freshwater white bass to have a greater ability to challenge a wide range of bacterial pathogens present in their environments compared to the striped bass. We challenged only one type of protozoan to piscidin activity assays in this study. With a wider range of protozoans, especially both fresh and saltwater obligate species, we may see a difference between the activities of piscidins 6 and 7, such that piscidin 7 is more active against marine compared to freshwater protozoans whereas piscidin 6 may be more effective in freshwater. This might be due to the wide range of protozoans that striped bass may encounter when moving between fresh and salt water and a better arsenal against protozoan pathogen attack may be optimal for this particular life history strategy.

It has been suggested that the diversification of the piscidin family is due to positive selection [60]. This is in accordance with gene diversification of other immune factors. Genetic diversification in the piscidin family also could be due to whole genome and/or individual gene duplication events known to have occurred during teleost evolution [61–63]. In our study, we found what could be products of such duplication events and that may have led to the current diversity of piscidins within each of the three proposed classes. Within each of these classes we have discovered what appear to be piscidin orthologs both within and between related fish species. These duplication events may have led to the subfunctionalization of some piscidin genes, as seen by the difference in gene expression of Class II piscidins 4 and 5, where the piscidin 5 locus is retained and expressed in white bass, however it is a regressed pseudogene in the striped bass (S2 Fig). This also may be the case with piscidin 7 in the striped bass, however a pseudogene of this particular locus has yet to be identified in white bass.

Neofunctionalization for example, could be indicated in the case of Class I piscidins 1 and 3, where the piscidin 1 forms from both striped bass and white bass have comparatively superior activities against certain species of bacteria assayed in this study. Also, orthologous piscidins in Classes I, II, and III have different anti-bacterial and anti-protozoan properties indicating neofunctionalization of piscidins in these groups. Although there are structural differences between the peptides of these orthologs, they share a high amino acid sequence identity to each other and there appears to be clear distinction in function. Further evidence supported by genomic synteny analysis of the piscidin loci in striped bass and white bass will be required to fully support this claim.

There is great variability in the mature and propeptide regions of the piscidins and those presented here may not be the entirety of this family of peptides found in the striped bass and white bass genomes. Each of the piscidin sequences described here were searched against a multi-tissue transciptomic dataset from striped bass and white bass [64]. One sequence in the striped bass transcriptome (comp42830_c0_seq1) was annotated in that study as pleurocidin, a member of the Antimicrobial 12 family. This sequence is identical to striped bass piscidin 7 described here. Thorough searching against the transcriptome sequence data identified all 5 of the striped bass piscidins described here, however only piscidin 6 was identified in the white bass transcriptome. An additional putative piscidin sequence was detected both in the striped bass genome sequence [65] and a striped bass transcriptome [64]. This gene codes for a 70 amino acid long pre-propeptide (MKCTAAFLVLSMVVFMAEPGECIWGMLIHGAIHTVGHLFNGLGKAKEQQEQQEQLDKRSIDYNPGRPHFH) and the top hit in a GenBank search matches the *Epinephelus coioides* piscidin 3 precursor (GenBank; AKA60776) with an e-value of 4e-26. No similar sequence was found in the white bass transcriptome. Further work will be required to confirm that this piscidin is functionally expressed and not an artifact of short read sequence assembly. Based on the predicted length and secondary structure of the mature peptide, this peptide appears to be similar to the Class I piscidins, however further study will be required to fully characterize its biological function and to confirm its class designation. This observation suggests that additional piscidin members will likely be described in these and other fish species in the future.

Because of the high variability in the mature and prodomain regions of the piscidins, the proposed piscidin classification scheme for striped bass and white bass may not apply to all fish species. Piscidins in Atlantic cod for example have varying functions based on slight differences in peptide structure [25]. Cod piscidin 1 is 22 amino acids long, and it has greater antibacterial activity than cod piscidin 2 and its splice variant (21 amino acids long), which have greater antiprotozoal activities. These three cod piscidins are similar in length to the Class I *Morone* piscidins, however the Class III piscidins in striped bass and white bass are the ones that exhibit anti-protozoal activity. Further analysis of piscidin activities will be necessary to identify if the piscidins from cod and other fishes fit into the classification scheme proposed here for striped bass and white bass. It does seem likely, though, that the diversity of piscidin forms found in other fish species would lead to diverse functions and potential of classification within species or genera.

In this study, we have expanded the number of known piscidins in fishes of genus *Morone* to nine, four more than had been previously published, and we have proposed dividing them into three classes based on peptide length, phylogenetic analysis, and functionality. Piscidin gene transcripts are differentially expressed in the tissues and each class varies in activity against bacterial versus protozoan targets, suggesting specialized functions for each piscidin class. Future work will be necessary to determine how the specificity of action relates to the structural properties of each piscidin class. Knowledge of piscidin function and activity may aid in the development of disease-resistant striped bass and white bass used in the production of hybrid striped bass. Furthermore, the piscidin expression and diverse spectrum of activity seen in this study could relate to the enhanced disease-resistance observed in the hybrid striped bass over either of the progenitor species. Future studies aimed at characterizing the rapid evolution of proteins involved in the innate immune system of diverse groups of fishes will further our understanding of protein and gene evolution.

## Supporting Information

S1 Fig Alignment of the striped bass and white bass piscidin 3 partial gene sequences. Two products were identified using hybrid striped bass genomic DNA as template along with primers specific for this gene. The larger, 723 bp, aligned with the sequence from striped bass (SB) while the smaller, 663 bp, aligned with the sequence from white bass (WB). The gray shaded boxes designate partial exonic regions; the unshaded region is an intron. The repeats of the smaller hybrid striped bass product (HSBS) and the white bass product were the same [(TC)_4_(TG)_12_(AGTG)_3_(TG)_2_]. Those of the larger hybrid striped bass product (HSBL) [(TG)_45_(AGTG)_2_(TG)_3_] and striped bass product [(TG)_34_(AGTG)_2_(TG)_3_] shared similar short tandem repeat composition, but differed in number of 5’ TG repeats (boxed regions).
(TIF)

S2 Fig Alignment of piscidin 5 mRNA and piscidin 5 partial genomic DNA sequences. Two products were identified in hybrid striped bass genomic DNA using primers for white bass piscidin 5. The start and stop codons are indicated by boxes. Introns are designated by gray boxes. Exonic regions which code for the mature peptide are in gray and not boxed. The genomic sequence in striped bass appears to be incomplete as cloning the region corresponding to the 5’ end of the putative gene transcript was unsuccessful thus indicating that the gene is not functional and does not encode a transcript with an open reading frame.
(TIF)

S1 Table Primers and TaqMan probes used in this study. (XLSX)

S2 Table Piscidin gene tissue expression in hybrid striped bass. (XLSX)
