# Supplementary figures and images for: A Diverse Family of Host-Defense Peptides (Piscidins) Exhibit Specialized Anti-Bacterial and Anti-Protozoal Activities in Fishes
Source: PLoS One. 2016 Aug 23;11(8):e0159423. doi: 10.1371/journal.pone.0159423 (PMC4995043; doi:10.1371/journal.pone.0159423)

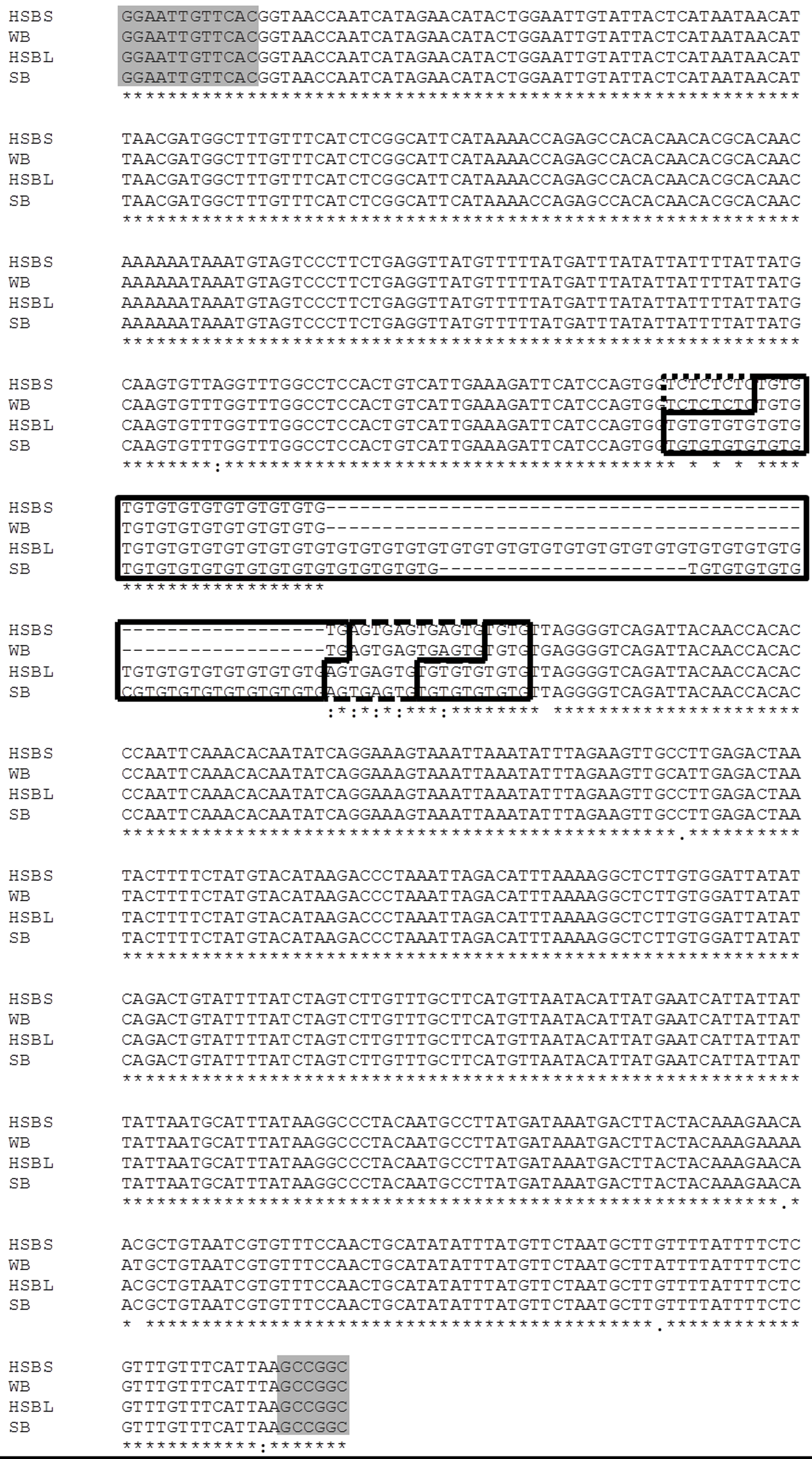

Supplement: S1 Fig — Two products were identified using hybrid striped bass genomic DNA as template along with primers specific for this gene. The larger, 723 bp, aligned with the sequence from striped bass (SB) while the smaller, 663 bp, aligned with the sequence from white bass (WB). The gray shaded boxes designate partial exonic regions; the unshaded region is an intron. The repeats of the smaller hybrid striped bass product (HSBS) and the white bass product were the same [(TC)4(TG)12(AGTG)3(TG)2]. Those of the larger hybrid striped bass product (HSBL) [(TG)45(AGTG)2(TG)3] and striped bass product [(TG)34(AGTG)2(TG)3] shared similar short tandem repeat composition, but differed in number of 5’ TG repeats (boxed regions). (TIF) [file pone.0159423.s001.tif]

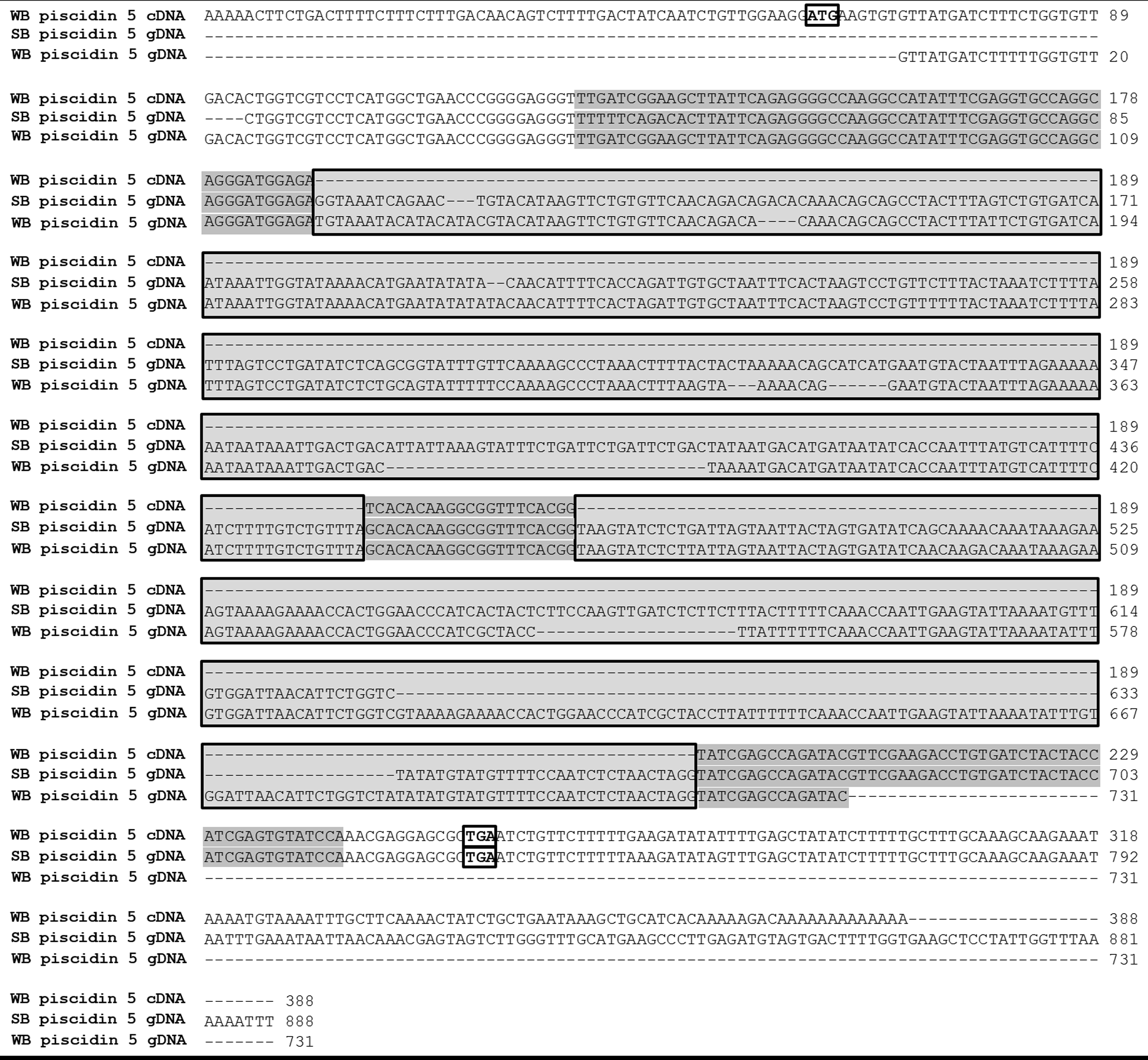

Supplement: S2 Fig — Two products were identified in hybrid striped bass genomic DNA using primers for white bass piscidin 5. The start and stop codons are indicated by boxes. Introns are designated by gray boxes. Exonic regions which code for the mature peptide are in gray and not boxed. The genomic sequence in striped bass appears to be incomplete as cloning the region corresponding to the 5’ end of the putative gene transcript was unsuccessful thus indicating that the gene is not functional and does not encode a transcript with an open reading frame. (TIF) [file pone.0159423.s002.tif]
